# Off to a Jump Start: Using Immersive Activities to Integrate Continuity Clinic and Advocacy

**DOI:** 10.1177/23821205211059652

**Published:** 2021-12-13

**Authors:** Kira Sieplinga, Emily Disbrow, Justin Triemstra, Monica van de Ridder

**Affiliations:** 1 Michigan State University College of Human Medicine, Pediatric Residency Spectrum Health/Helen DeVos Children’s Hospital, Grand Rapids, MI, USA; 2 Michigan State University College of Human Medicine, Pediatric Residency Sparrow Hospital, Lansing, MI, USA

**Keywords:** Advocacy, immersion, socio-constructivism, CHAMP mapping tool

## Abstract

**BACKGROUND:**

Training in advocacy is an important component of graduate medical education. Several models have been implemented by residency programs to address this objective. Little has been published regarding application of immersive advocacy activities integrated into continuity clinic.

**OBJECTIVE:**

To create an Integrated Community Health and Child Advocacy Curriculum (ICHCA) by integrating advocacy activities that were immersive and contextualized in a continuity clinic setting and to familiarize interns with continuity clinic immediately at the beginning of their training.

**METHODS:**

We utilized a socio-constructivist lens, Kern's Six-step curriculum development and a published curriculum mapping tool to create the curriculum. Twenty residents completed ICHCA in 2019. Evaluations from key stakeholders including participants, support staff and attendings were analyzed on four levels of Kirkpatrick's model. We compared results before intervention, immediately following intervention and ten months following intervention.

**RESULTS:**

We demonstrated improvement in learner satisfaction, knowledge and behaviors with respect to advocacy in the clinical environment. Response rate was 70% (7/10) for attendings, 75% for support staff (15/20) and 72.5% for residents (29/40). Our intervention was feasible, no cost, and required no additional materials or training as it relied on learning in real time.

**CONCLUSIONS:**

An integrated advocacy curriculum utilizing the mapping tool for curricular design and evaluation is feasible and has value demonstrated by improvements in reaction, knowledge, and behaviors. This model improves understanding of social responsibility and can be implemented similarly in other residency programs.

## Introduction

“If physicians simply patch up those who are sick … rather than pushing for the changes needed to actually prevent [illness], we will not have as great an impact on health. If we are going to advocate successfully for those changes, then such advocacy must become a core value that we teach … in residency.”^1^ Joshua Freeman described health advocacy's potential impact on curricular development and inspired the authors to revisit advocacy in our local institution.

### Overview of health advocacy in pediatric medicine

Over the past two decades, American Graduate Medical Education has embraced advocacy as a core educational value. For example, in 2001, the American Medical Association adopted a “Declaration of Physician Responsibility: Medicine's Social Contract with Humanity” which states that physicians should commit to “advocate for social, economic, educational, and political changes that ameliorate suffering and contribute to human well-being.”^2^ The Accreditation Council for Graduate Medical Education (ACGME) has recognized training in advocacy as an objective of graduate medical education.^3^ In 2005, the American Academy of Pediatrics Community Pediatrics Training Initiative (CPTI) developed a set of goals and objectives for pediatric residents in community health and advocacy.^4^ Building on this work, the Community Health and Advocacy Milestones Profile (CHAMP) was developed.^5^ This curriculum development tool maps pediatric milestones to the thirty-six CPTI objectives in eight content areas.^6^ Multiple programs have utilized this tool to implement advocacy curriculum.^6,7^ Little has been published regarding the immediate application of an advocacy curriculum into the primary context of pediatric residents work environment: the continuity clinic. The continuity clinic experience is an ACGME requirement for many residency programs including general pediatrics. Residents are required to rotate in the same location for 36 separate clinic half days in each year of training in order to promote longitudinal relationships with patients.

Several ambulatory rotations have been integrated with an advocacy curriculum at one center^8^ and the continuity clinic setting has been studied as a venue for specific advocacy-related activities.^9^ In 2017, Howell et al. published a systematic review using thematic analysis to evaluate published advocacy curricula in graduate medical education. Of the 38 articles included for qualitative analysis, teaching methodologies varied; however, none described rotations that focused on both improving continuity clinic experience and improving advocacy clinic.^10^ Specifically, no publications describe an integrated rotation solely focused on continuity clinic and advocacy: education that is by definition immersive and contextualized or “meaning making” as the socio-constructivists would describe.^8,10^

### Socio-constructivism

From a socio-constructivist perspective, learning is seen as an activity that happens not only in a formal classroom context, but it is part of our everyday experiences.^11^ Learners engaged in contextualized activities connect ‘new’ knowledge to previously acquired knowledge.^12^ In addition, social context and culture play an important role in the learning process as ‘meaning making’ takes place through interaction with community, activities and culture.^12–15^ One method to create learning context is through ‘immersion’ which is defined as “to place someone or something into a state of being.”^16^ In medical education, the concept of immersion has been explored both with respect to the continuity clinic experience^17^ and in advocacy curricula.^18,19^

### Local advocacy and clinic context

At our institution, a required month-long advocacy rotation had existed for approximately ten years and included primarily self-driven independent activities. Despite the autonomy, residents were dissatisfied with the experience and used descriptors including: “random, disjointed, and disorganized”^20^ in surveys and end-of-rotation evaluations. Assignments were not evaluated or debriefed and there was no context to immediately apply skills and knowledge. Residents completed this rotation in isolation in their second year of residency. An unpublished needs-assessment among residents (n = 35) revealed 54% perceived the established advocacy rotation was not congruent with their personal definition of “advocacy” and 70% were dissatisfied with the rotation. Similarly, 60% stated they were not emotionally fulfilled with the rotation, highlighting the need for change. Along with this model of advocacy training, residents in our institution followed a traditional half-day per week continuity clinic schedule over the course of three years.

With a socio-constructivist immersive lens, and through use of the CHAMP mapping tool, we sought to improve *both* the clinic and advocacy experience. Providing a “jump start,” we integrated advocacy training into a continuity clinic rotation at the start of the pediatric intern's academic year. Kirkpatrick's model was used for evaluation, and we hypothesized that this curriculum would show improvements on all levels.^21^ In addition, we explored whether the objectives linked to the CHAMP mapping tool could be used to create activities and evaluate behaviors.

## Methods

### Curriculum development

**Figure 1.**
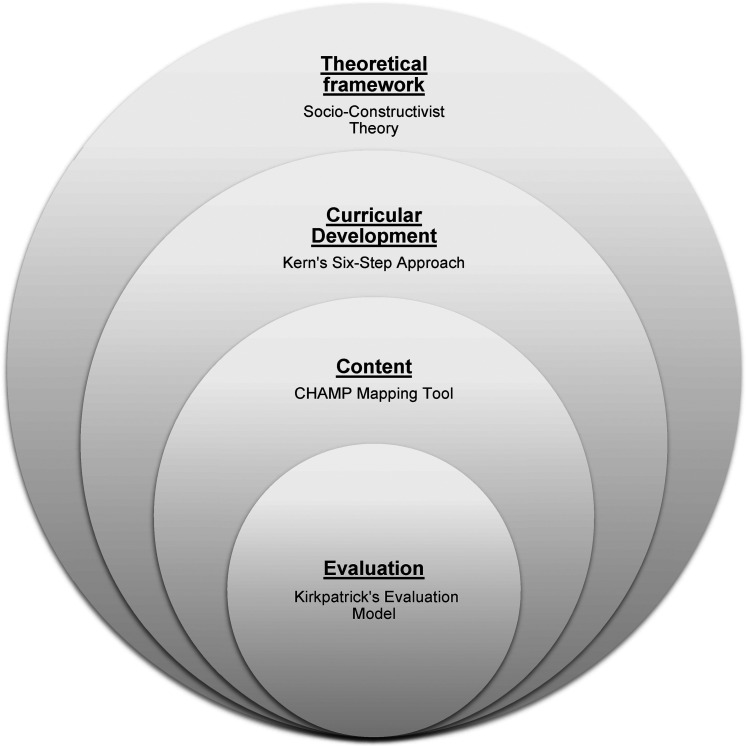
Integrated community health and child advocacy curriculum (ICHCA): frameworks and tools used for curriculum design and evaluation.

**Figure 2.**
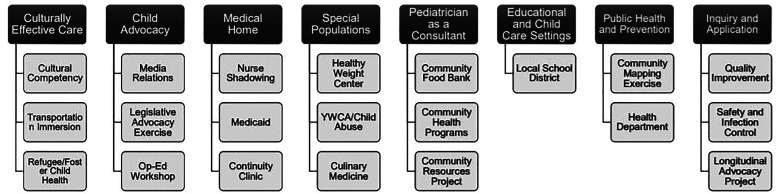
Integrated community health and child advocacy curriculum (ICHCA): informed by community health and advocacy milestones profile (CHAMP) mapping tool. Each header represents one element of the mapping tool. The activities listed below each header represent experiences identified by local experts contextualized in our midwest urban community.

Kern's Six-step approach^22^ and the CHAMP mapping tool served as frameworks for curriculum development (Figure 1). The new *Integrated Community Health and Child Advocacy Curriculum (ICHCA)* became known as “clinic immersion.” Stakeholders including residents, support staff and attending academic pediatricians utilized the CHAMP mapping tool to create curricular activities that were of no cost, feasible and easily accessible in our Midwest urban setting (Figure 2).

**Figure 3.**
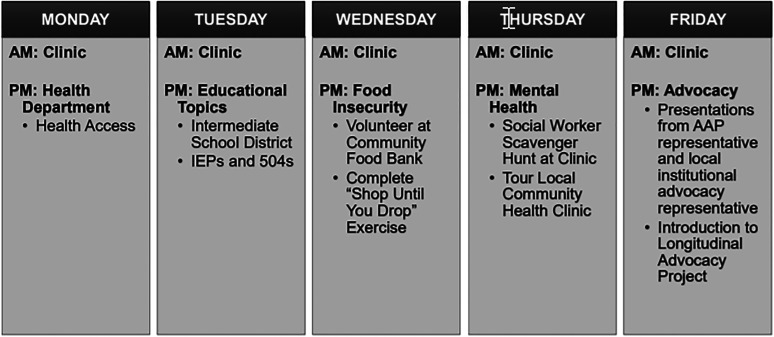
Integrated community health and child advocacy curriculum (ICHCA) representative weekly calendar. Criteria for experience selection: expert agreement, feasibility, no cost, alignment with socioconstructivist lens.

In 2019, twenty interns experienced ICHCA during either block one or two of their year. The 28-day blocks were chosen intentionally at the start of the academic year and the twenty interns represented their entire class with ten interns assigned to block 1 and ten interns assigned to block 2 with similar experiences. No control group was formed due to the complexity of running two simultaneously curricula. Interns were the only scheduled residents in the academic pediatric clinic in the mornings and were precepted by attending pediatricians. Senior residents were scheduled for the afternoons. Each week, the number of patients scheduled was gradually increased, and core general pediatric topics were introduced. In the afternoons, interns were assigned experiences informed by the CHAMP mapping tool (see Figure 3 for a sample week). Because ICHCA utilized experiences with a socio-constructivist lens, no additional faculty or staff training was needed. Instead, connections with community agency leaders were made to provide educational activities. In all cases, these leaders provided time voluntarily, typically hosted interns on-site and expressed a strong desire to make connections with the pediatric residents. In block three following successful completion of ICHCA, interns return to a more traditional continuity clinic schedule of one half-day per week during their assigned clinical rotations.

### Curriculum evaluation methods

Kirkpatrick's levels of evaluation informed the curricular assessment.^21^ (See Table 1) We anonymously assessed the evaluations of three distinct groups of stakeholders: residents, attending academic pediatricians and clinical support staff members (medical assistants, nurses, social workers and clinic administrators). Nurses are known to be effective evaluators of resident milestones and competencies.^23^ The role of nurses and other clinical staff in curricular evaluation is less well established but aligns with a socio-constructivist framework.

**Table 1.** Integrated community health and child advocacy curriculum (ICHCA) curriculum evaluation.

Kirkpatrick's levels	Evaluators^a^	Outcomes*
Reaction	R	Satisfaction with rotation increased 49 to 93%*
		Confidence in knowledge of community resources increased from 38% to 60%*
		Confidence in knowledge of health disparities and types of patients in the community increased*
	SS	Intern's comfort in clinic was achieved more quickly
	A	Intern's comfort in clinic was achieved more quickly (three months vs six months pre-ICHCA)*
Learning	R	Residents had exposure to 13/16 clinical skills one month after ICHCA versus ten months into the traditional curriculum
	SS	Reported no change in frequency of answering questions or providing corrections
	A	Perception of intern's vaccine and screening accuracy trended towards improvement
Behaviors	SS	Reported no change in their perception of how frequently families have questions after an intern has completed a visit
	A	Ability of the intern class to implement CHAMP curricular objectives trended toward improvement (see Table 2)
Results	Admin^b^	No significant variation in clinic relative value units (RVUs) both pre and post-ICHCA

*Indicates statistical significance (*P* < .05).

^a^ Response rates: Residents [R] (70% pre-ICHCA – 14/20; 75% post-ICHCA – 15/20); Support Staff [SS] (75% pre and post-ICHCA – 15/20); Attendings [A] (70% pre and post-ICHCA – 7/10).

^b^ Administrator (Author JT & Independent Analyst).

**Table 2.** Community health and advocacy milestones profile (CHAMP): number of attendings (*n* = 7) who rated the intern class as “good, very good or excellent” versus “fair and poor” for each CHAMP objective (full objectives in Supplementary Digital Content).

Selected CHAMP objectives	Pre-ICHCA	Post-ICHCA
Culturally effective care #4 (identify, analyze, describe)	3	5
Child advocacy #1 (identify, discuss)	3	5
Child advocacy #2 (formulate)	2	4
Medical home #2 (identify)	3	5
Medical home #5 (describe, outline)	3	5
Special populations #1 (identify)	5	7
Special populations #3 (demonstrate)	2	4
Pediatrician as a consultant #1 (identify)	2	4
Educational and child care settings #1 (promote)	2	4
Educational and child care settings #2 (explain)	3	5
Public health and prevention #4 (identify, describe)	3	4
Public health and prevention #5 (describe, discuss)	4	4

*Reactions* were assessed by querying satisfaction with the curriculum, confidence in knowledge and overall comfort in the clinic setting through use of a homegrown survey (See Appendices). Residents used a continuous visual analog scale to rate their confidence in knowledge addressing common outpatient clinic visits, community resources and health disparities. Support staff were queried about how quickly the intern class was comfortable with most types of patient visits.

*Learning* was assessed by asking residents whether they had exposure to 16 key activities. They responded dichotomously using a “yes” or “no” checklist. In addition, support staff and attendings were asked how often they need to make a correction to a resident plan (see Appendices).

*Behaviors* were assessed by attendings using the CHAMP mapping tool. Utilizing local expert opinion, we selected 12 objectives from the 8 content areas listed on the CHAMP mapping tool as they related directly to curricular activities provided during the rotation. Attending pediatricians were asked to think of the intern class as a group and then asked to rate the group as to how well they were able to implement the CHAMP objective. Language from the CHAMP objectives were used verbatim and a five-point Likert scale was used rating the intern group from poor to excellent for each objective. The newly created evaluation tool was taught and demonstrated to general pediatric attendings prior to its use.

In addition, behaviors were assessed by support staff. Support staff were asked how frequently families still have questions after a visit with a resident.

Because this was a pilot single site study, we assessed clinic relative value units (RVUs) to assess the *results* and potential financial impact of the curriculum. We compared the total RVUs from the continuity clinic location for July and August of 2018 (pre ICHCA implementation) to July and August of 2019 (post ICHCA implementation). Values were retrieved and compared by one of the authors and an independent analyst.

Clinical support staff (n = 20) and attendings (n = 10) were surveyed at the end of the academic year prior to ICHCA and at the end of the academic year following ICHCA. Interns who had never experienced ICHCA prior to its implementation were surveyed at the end of their intern year (n = 19) and the interns who participated in ICHCA were surveyed both immediately and ten months following completion (n = 20).

Pearson's chi-squared test was used when comparing responses and significance was assessed at *P* < .05. The project was reviewed by the Spectrum Health IRB and deemed exempt.

## Results

In the three groups that were surveyed pre- and post-ICHCA, we demonstrated results at multiple levels of evaluation (see these summarized in Table 1). Response rate for residents were 70% pre-ICHCA (14/20) and 75% post-ICHCA (15/20). Support staff response rate was 75% both pre and post-ICHCA (15/20) and response rate for attendings was 70% both pre and post-ICHCA (7/10). We have organized the results by respondants.

### Resident participant results

#### Reactions

Satisfaction of the advocacy rotation increased from 49 to 93% between pre-intervention and post-intervention cohorts. 100% of the post-intervention cohort interns stated the rotation was consistent with their personal definition of advocacy and was ‘emotionally fulfilling’. Confidence in knowledge of community resources increased 38% to 60% (*P* = .001) immediately following the rotation. Confidence in knowledge of the types of patients served by the clinic and health disparities was sustained at time point 2 (10 months into the intern year) 66% to 75% (*P* = .011) and 66% to 78% (*P* = .009), respectively.

#### Learning

With respect to speed to acquisition of skills and learning within the new curriculum, we determined that by the end of immersion (one month of clinic experience), post-intervention cohort interns had equal statistical exposure to 13/16 clinical skills when compared to pre-intervention cohort interns ten months into their intern year. By the end of immersion, the post-intervention cohort had not had equal exposure to referring to a subspecialist, ordering an imaging study, or making a referral to Early On. However, when the post-intervention cohort was re-queried ten months into their intern year, all clinical exposure was equal to their colleagues from the pre-intervention cohort.

### Clinical support staff

#### Reactions

Support staff noted increased speed to comfort post-intervention with 63% of support staff endorsing comfort in clinic three months into the year post-intervention versus 50% endorsing this prior to intervention (*P* = .328). Comments from support staff included: “I think that the immersion this year helped very much with helping the interns to become familiar with the clinic and the processes of the clinic.” and “I feel this group of interns rapidly became familiar with clinic after their first 2 months in clinic.”

#### Learning/behaviors

Support staff did not report a statistically significant change in a positive or negative direction with respect to speed, efficiency or the need to recommend changes in the post-intervention group.

### Academic general pediatric faculty

#### Reactions

All attendings rated interns as “comfortable with most types of patient visits” by three months into the year post-intervention as opposed to 6 months into the year pre-intervention (*P* = .008) Comments from attendings regarding this experience included: “Our interns were much more comfortable in clinic.” and “I saw a great change! I loved the immersion for the social support/camaraderie that it prompted, and [I noted] much better medical knowledge/comfort with clinic.”

#### Learning

Attending physicians *trended* towards improvement with respect towards making recommendations or changes regarding an intern's plans with respect to vaccines and required screening but this did not reach statistical significance (*P* = .192)

#### Behavior

Similar to the support staff, attending physicians did not report a change in speed or efficiency post-curricular intervention. Attendings reported an overall trend towards IMPROVEMENT in clinical behaviors in competencies established by the CHAMP mapping framework (see Figure 3). We asked the attendings to think of the intern class as a group and then specifically asked them how well they were able to implement each of the competencies on a five-point ranging from poor to excellent. All competencies showed improvement after implementation of the new curriculum (with the exception of Public Health & Prevention #5). However, competencies focusing on “identification” showed more improvement than those asking interns to “demonstrate,” “formulate” or “outline” specific advocacy concepts. None of the change reached statistical significance due to convenience sample size of only 7 attendings in this single site pilot study.

#### Results

There was no significant variation in clinic RVU with implementation of ICHCA.

## Discussion

To our knowledge, literature examining the systematic integration of continuity clinic and advocacy experiences has not occurred. This pilot study began to explore whether embedding an advocacy curriculum informed by the CHAMP mapping tool into a residency continuity clinic block placed intentionally at the beginning of intern year could improve both continuity clinic and advocacy experience. Our study demonstrates the success of utilizing a socio-constructivist approach and uses the CHAMP mapping tool to link clinical experiences (morning clinic), advocacy knowledge and immersive activities in community settings.

Ultimately, we strove to move activities that had classically occurred in classroom settings, to real-world, contextualized community experiences.^11^ We did this to ensure residents were actively engaged in these experiences and would be able to contextualize their learning into their clinical activities. Because they were simultaneously learning in the clinical setting, we believe our residents were able to connect their new knowledge of local community resources with previously acquired general knowledge on community health advocacy and health equity and directly apply them to patient care. This was demonstrated by the results in Kirkpatrick levels one, two and three as shown by improvement in intern satisfaction with new curriculum, increased confidence in knowledge (community health resources, community served, and health disparities, earlier clinical experiences), and faculty perspective on behaviors as measured by the CHAMPS evaluation tool. Additionally, clinical exposure occurred much more quickly as interns were now exposed to the same amount of clinical skills in one month that the pre-intervention cohort were exposed to at ten months into residency training.

Furthermore, positive trends were also seen in the faculty's perspective on intern's knowledge (as measured by need to suggest change) and implementation of CHAMP mapping tool objectives. Utilizing the CHAMP mapping tool as an evaluation tool may be useful for evaluation of future advocacy curricula. In this initial iteration, we triangulated our evaluation by incorporating evaluations of the curriculum by learners, faculty, and clinical support staff members. We understand the positive effect of simply intensifying the continuity clinic into a one month “jump start” rotation could lead to improved recognition and knowledge of community health resources, however, this in it of itself, is an objective of the curriculum and something other residency programs should consider.

Finally, although not specifically queried, the rotation served as an enhanced orientation for incoming interns and promoted bonding and wellness among the intern cohort and support staff.

Limitations of this study include single site and small faculty evaluation numbers which limits both the significance of the CHAMP evaluation tool. In addition, more work needs to be done to demonstrate validity of the CHAMP tool for evaluation purposes in future cycles. Our comparison groups (pre-ICHCA and post-ICHCA) were both residents in equal levels of training; however significant differences between the two groups should be noted. The initial momentum and enthusiasm for a change and a new curriculum in and of itself could influence results. In addition, the curriculum is complex and addresses two distinct objectives: continuity clinic and advocacy reactions, learning and behaviors making the effects difficult to tease out.

Moving forward, we will incorporate the assessment of the curriculum from the residents, faculty and clinic support staff to improve the curriculum, while targeting Kirkpatrick level 4 by evaluating direct use of community resources or resident engagement in enhanced advocacy projects. Our limited assessment of RVU trends were from two time points and thus causation cannot be claimed. Further attention should be directed towards financial impact or potential improvement given early introduction to clinical skills as we initially hypothesized that a clinical immersion could lead to improved overall RVUs per intern given their speed to achieve full clinic schedules.

## Conclusion

An integrated advocacy curriculum, utilizing the CHAMP mapping tool in a continuity clinic setting is feasible and has perceived value as demonstrated by improvements in satisfaction, knowledge, and behaviors. Training residents to have improved knowledge of community health resources and health disparities improves their understanding of social responsibility and will improve the care they provide to their patients. The methods used in curricular design and evaluation could be applied in other graduate medical education programs across multiple fields. We hope that through this immersive experience, advocacy will be acquired as a core value of residents who will potentially influence the health of community beyond “patching up those who are sick.”
